# Ultrasound prediction of adverse perinatal outcome at diagnosis of late‐onset fetal growth restriction

**DOI:** 10.1002/uog.23714

**Published:** 2022-03-03

**Authors:** A. Dall'Asta, T. Stampalija, F. Mecacci, M. Minopoli, G. B. L. Schera, G. Cagninelli, C. Ottaviani, I. Fantasia, M. Barbieri, F. Lisi, S. Simeone, T. Ghi, T. Frusca

**Affiliations:** ^1^ Department of Medicine and Surgery, Unit of Surgical Sciences, Obstetrics and Gynecology University of Parma Parma Italy; ^2^ Unit of Fetal Medicine and Prenatal Diagnosis Institute for Maternal and Child Health IRCCS Burlo Garofolo Trieste Italy; ^3^ Department of Medicine Surgery and Health Sciences, University of Trieste Trieste Italy; ^4^ Department of Biomedical, Experimental and Clinical Sciences, Division of Obstetrics and Gynecology University of Florence Florence Italy

**Keywords:** cerebroplacental ratio, elective delivery, perinatal complications, umbilical artery Doppler, uterine artery Doppler

## Abstract

**Objective:**

To evaluate the relationship between Doppler and biometric ultrasound parameters measured at diagnosis and perinatal adverse outcome in a cohort of late‐onset growth‐restricted (FGR) fetuses.

**Methods:**

This was a multicenter retrospective study of data obtained between 2014 and 2019 including non‐anomalous singleton pregnancies complicated by late‐onset FGR (≥ 32 weeks), which was defined either as abdominal circumference (AC) or estimated fetal weight (EFW) < 10^th^ percentile for gestational age or as reduction of the longitudinal growth of AC by over 50 percentiles compared to ultrasound scan performed between 18 and 32 weeks of gestation. We evaluated the association between sonographic findings at diagnosis of FGR and composite adverse perinatal outcome (CAPO), defined as stillbirth or at least two of the following: obstetric intervention due to intrapartum fetal distress, neonatal acidemia, birth weight < 3^rd^ percentile and transfer to the neonatal intensive care unit (NICU).

**Results:**

Overall, 468 cases with complete biometric and umbilical, fetal middle cerebral and uterine artery (UtA) Doppler data were included, of which 53 (11.3%) had CAPO. On logistic regression analysis, only EFW percentile was associated independently with CAPO (*P* = 0.01) and NICU admission (*P* < 0.01), while the mean UtA pulsatility index (PI) multiples of the median (MoM) > 95^th^ percentile at diagnosis was associated independently with obstetric intervention due to intrapartum fetal distress (*P* = 0.01). The model including baseline pregnancy characteristics and the EFW percentile was associated with an area under the receiver‐operating‐characteristics curve of 0.889 (95% CI, 0.813–0.966) for CAPO (*P* < 0.001). A cut‐off value for EFW corresponding to the 3.95^th^ percentile was found to discriminate between cases with and those without CAPO, yielding a sensitivity of 58.5% (95% CI, 44.1–71.9%), specificity of 69.6% (95% CI, 65.0–74.0%), positive predictive value of 19.8% (95% CI, 13.8–26.8%) and negative predictive value of 92.9% (95% CI, 89.5–95.5%).

**Conclusions:**

Retrospective data from a large cohort of late‐onset FGR fetuses showed that EFW at diagnosis is the only sonographic parameter associated independently with the occurrence of CAPO, while mean UtA‐PI MoM > 95^th^ percentile at diagnosis is associated independently with intrapartum distress leading to obstetric intervention. © 2021 The Authors. *Ultrasound in Obstetrics & Gynecology* published by John Wiley & Sons Ltd on behalf of International Society of Ultrasound in Obstetrics and Gynecology.

**CONTRIBUTION**

*What are the novel findings of this work?*

The findings of this study show that, at the time of diagnosis of late‐onset fetal growth restriction (FGR), estimated‐fetal‐weight percentile is the only ultrasound parameter associated independently with composite adverse perinatal outcome, defined as stillbirth or at least two of the following: obstetric intervention due to intrapartum fetal distress, neonatal acidemia, birth weight < 3^rd^ centile and transfer to the neonatal intensive care unit, while uterine artery pulsatility index multiples of the median > 95^th^ percentile is associated with intrapartum fetal distress leading to obstetric intervention.

*What are the clinical implications of this work?*

In this cohort study, including non‐anomalous singleton pregnancies defined as late‐onset FGR according to the study inclusion criteria, we observed a low frequency of maternal, perinatal and postnatal complications. Our data can be used for antenatal counseling of prospective parents.

## INTRODUCTION

Fetal growth restriction (FGR) is a complex and multifactorial disorder affecting fetal development, which represents currently one of the leading causes of perinatal mortality and morbidity, including iatrogenic preterm delivery^1^, ^2^. It is also known to be associated with an increased risk of suboptimal neurodevelopmental outcome^3^ and long‐term morbidity, including cardiovascular disease^4^, ^5^.

According to the Delphi consensus criteria, FGR is differentiated into early‐ and late‐onset phenotypes, depending on whether it is identified before or after 32 weeks' gestation^6^. Uteroplacental insufficiency is acknowledged to represent the major determinant of impaired fetal growth across gestation^7^, ^8^, ^9^. However, available evidence has shown that early‐ and late‐onset FGR types are characterized by different clinical presentation. The former is a rare condition, associated commonly with hypertensive disorders of pregnancy (HDP) and a high risk of preterm delivery^10^, ^11^, ^12^, ^13^, while the latter is more common but difficult to discriminate from non‐pathological smallness^14^.

Except for delivery, there is no effective treatment to reverse the course of FGR. However, while evidence for management of FGR with onset between 26 and 32 weeks' gestation has emerged from the TRUFFLE study^10^, ^11^, ^12^, ^13^, to date, there has been no grade‐A evidence supporting the monitoring strategy and timing of delivery in late‐onset FGR. Furthermore, there are limited data on the occurrence of perinatal complications when a diagnosis of late‐onset FGR is made. As such, interest has increased in quantitative evaluation of estimated fetal weight (EFW)^15^ and Doppler assessment of the fetal hemodynamic response to hypoxemia, evaluated by means of cerebroplacental ratio (CPR) or umbilical‐to‐cerebral ratio, and the placental impedance to uterine perfusion, which can be assessed using uterine artery (UtA) Doppler.

Several studies have demonstrated a relationship between sonographic indicators of placental insufficiency, cerebral blood flow redistribution and adverse perinatal outcome in late‐onset FGR^16^, ^17^, ^18^, ^19^, ^20^. Furthermore, isolated severe FGR, i.e. abdominal circumference (AC) or EFW < 3^rd^ percentile, has also been proposed as an additional risk factor for fetal or neonatal complications^21^, ^22^, ^23^. On these grounds, the latest and widely agreed definition of late‐onset FGR includes not only a biometric percentile threshold, but also sonographic indicators of reduced longitudinal growth and cerebral redistribution^6^.

The recently published data of the prospective observational feasibility study conducted by the TRUFFLE group has led to the identification of thresholds for umbilical and cerebral Doppler values which correlate most strongly with adverse outcome^16^. These cut‐off values are being evaluated in a randomized controlled trial (ISRCTN: 76016200) investigating the role of Doppler assessment of the umbilical‐to‐cerebral ratio in monitoring and timing of delivery in late‐onset FGR^16^, ^24^, in which late‐onset FGR is defined as either EFW or AC < 10^th^ percentile or as decrease in AC of > 50 percentiles from ultrasound scan at 18–32 weeks' gestation. Using the same diagnostic criteria adopted by the TRUFFLE group, in this study we aimed to evaluate the relationship between Doppler and biometric ultrasound parameters measured at diagnosis and adverse outcome at birth within a selected cohort of late‐onset FGR fetuses.

## METHODS

This was a retrospective study conducted at three tertiary academic units in Italy (University of Parma, Parma, Italy; University of Florence, Florence, Italy; and University of Trieste, Trieste, Italy), including a consecutive series of non‐anomalous singleton pregnancies referred for expert ultrasound between 32 + 0 and 36 + 6 weeks of gestation due to suspected FGR, between January 2014 and December 2019. All pregnancies were dated based on first‐trimester crown–rump length.

All non‐anomalous singleton pregnancies referred between 32 + 0 and 36 + 6 weeks due to suspected FGR were screened. Diagnosis of late‐onset FGR was made based on EFW or AC < 10^th^ percentile or decrease in AC of > 50 percentiles from the ultrasound scan performed between 18 and 32 weeks. For each pregnant woman, information concerning fetal biometry and maternal Doppler assessment at diagnosis (i.e. mean UtA pulsatility index (PI) and fetal (i.e. umbilical artery PI, middle cerebral artery (MCA) PI and the CPR) was recorded. EFW was computed by means of the Hadlock IV formula^25^, while Doppler assessment was performed following the recommendations of the International Society of Ultrasound in Obstetrics and Gynecology^26^. In order to adjust for gestational age, EFW percentile was considered, while Doppler parameters were converted into multiples of the median (MoM).

Cases were excluded in the event of missing data for umbilical artery, MCA and UtA Doppler at the time of diagnosis of late‐onset FGR or perinatal outcome and in the event of postnatal diagnosis of malformation, chromosomal abnormality, genetic syndrome or congenital infection.

As per management protocol, which was consistent in the three participating centers, follow‐up scans were arranged every 2 weeks in the absence of signs of cerebral redistribution, defined as CPR > 5^th^ percentile, on a weekly basis in the case of CPR < 5^th^ percentile or UtA‐PI > 95^th^ percentile and on alternate days in the case of umbilical artery PI > 95^th^ percentile. Induction of labor was recommended between 36 and 37 weeks in the case of umbilical artery PI > 95^th^ percentile, at 37 weeks in the case of EFW or AC < 3^rd^ percentile, between 37 and 38 weeks if signs of redistribution were noted (i.e. CPR < 5^th^ percentile) and at 39–40 weeks in the absence of any of those risk factors for adverse perinatal outcome. The monitoring strategy also included cardiotocography (CTG), which was performed at each assessment. The finding of absent or reversed end‐diastolic (ARED) flow in the umbilical artery and spontaneous repetitive decelerations on CTG represented indication for immediate delivery by Cesarean section. Antenatal steroids for lung maturation were administered in all cases delivering before 34 + 6 weeks^27^. Pre‐eclampsia represented an indication for delivery at 37 weeks, or 34 weeks in the presence of clinical signs of severity of the disease^28^.

In all cases referred for labor induction, cervical ripening was promoted either by means of a cervical ripening balloon or by vaginal administration of a slow‐release prostaglandin‐E2 pessary, which was followed by oxytocin infusion if the onset of labor did not occur. Cesarean section was performed electively in the presence of ARED flow in the umbilical artery, spontaneous repetitive decelerations on CTG monitoring or other obstetric indications. The clinicians responsible for the intrapartum care were not blinded to the antepartum findings. The diagnosis of intrapartum fetal distress was defined by the physician in charge of the patient care based on abnormal CTG tracing according to the FIGO classification system^29^. As per standard practice, the paired analysis of the cord gases was performed at birth according to the recommendations of the American College of Obstetricians and Gynecologists^30^.

Information on maternal demographics and peripartum and neonatal outcome were collected from patient case notes. Neonatal outcome was assessed by examining birth weight and birth‐weight percentile corrected for gender^31^, cord arterial pH and base excess at delivery, Apgar score at 5 min, need for resuscitation at birth, need for respiratory support at birth, neonatal jaundice (serum bilirubin > 20.6 mg/dL (350 µmol/L))^32^, neonatal hypoglycemia (two consecutive whole‐blood glucose measurements by glucometer or one plasma glucose measurement < 3.3 mmol/L)^33^, need for admission to neonatal intensive care unit (NICU) and length of neonatal admission.

The primary aim of this study was to evaluate the relationship between sonographic parameters measured at diagnosis and the occurrence of CAPO, defined as stillbirth or at least two of the following: obstetric intervention due to intrapartum fetal distress, neonatal acidemia, defined by umbilical artery pH^34^ < 7.10, birth weight < 3^rd^ percentile and transfer to NICU. Additionally, neonatal hypoglycemia, defined by two consecutive whole‐blood glucose measurements by glucometer or one plasma glucose measurement < 3.3 mmol/L^33^, neonatal hyperbilirubinemia/jaundice, defined by serum bilirubin above 20.6 mg/dL (350 µmol/L)^32^, need for respiratory support at birth and the length of neonatal admission were evaluated.

Ethical approval for collection of the data for this study was granted by the local ethics committee of the involved centers.

Statistical analysis was performed using SPSS, version 20 (IBM Inc., Armonk, NY, USA). Data are shown as mean ± SD, *n* (%) or median (range). Categorical variables were compared using the chi‐square or Fisher's exact test. Comparison of continuous variables was performed using the independent‐samples *t*‐test and two‐tailed *t‐*test. Logistic regression analysis was used to control for potential confounding variables and receiver‐operating‐characteristics (ROC)‐curve analysis was performed in order to evaluate the accuracy of the prediction of primary outcome. The method of DeLong *et al*.^35^ was used for comparison of ROC curves. *P* < 0.05 was considered to be statistically significant. This study was conducted following the STROBE guidelines^36^.

## RESULTS

**Figure 1:**
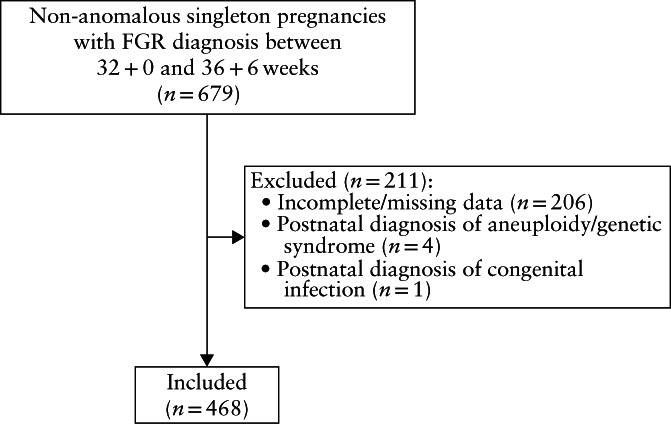
Flowchart summarizing inclusion of cases with suspected fetal growth restriction (FGR).

Overall, 679 cases (253 from the University of Parma, 258 from the University of Florence and 168 from the University of Trieste) with late‐onset FGR defined according to the study inclusion criteria were retrieved, of which 468 were included for data analysis following evaluation of the exclusion criteria (Figure 1). Demographic features and perinatal outcome of the included cases are summarized in Table 1. One case of stillbirth was recorded. Obstetric intervention due to intrapartum fetal distress was performed in 41 cases, which accounted for 8.8% of all deliveries and 11.6% of deliveries when cases with elective Cesarean section were excluded. Over 60% of neonates had birth weight < 10^th^ percentile, with no difference in terms of adverse outcome between those with birth weight above *vs* below the 10^th^ percentile threshold (Table S1). Neonatal acidemia, NICU admission and CAPO were recorded in six (1.7%), 108 (23.1%) and 53 (11.3%) cases, respectively, while neonatal hypoglycemia and need for respiratory support at birth were recorded in 91 (19.4%) and 32 (6.8%) cases, respectively (Table 1).

**Table 1:** Demographic characteristics and perinatal outcome of 468 cases with late‐onset fetal growth restriction (FGR)

Characteristic	Value
Maternal age (years)	32.6 ± 5.7
BMI at booking (kg/m^2^)	22.1 ± 4.1
BMI at delivery (kg/m^2^)	26.3 ± 4.3
Ethnicity	
Caucasian	402 (85.9)
African	20 (4.3)
Asian	32 (6.8)
Other	14 (3.0)
Nulliparous	328 (70.1)
Smoker	48 (10.3)
Comorbidity	
HDP	28 (6.0)
DM/GDM	30 (6.4)
Autoimmune disorder	19 (4.1)
GA at diagnosis (weeks)	34 + 2 ± 1 + 4
GA at delivery (weeks)	38 + 2 ± 1 + 4
Delivery < 37 weeks	70 (15.0)
Delivery < 34 weeks	6 (1.3)
Mode of delivery	
SVD	294 (62.8)
ID due to suspected IFD	5 (1.1)
CS due to suspected IFD	36 (7.7)
Elective CS due to FGR	38 (8.1)
Elective CS non‐FGR	78 (16.7)
ID due to dystocia	7 (1.5)
CS due to dystocia	10 (2.1)
Male neonate	217 (46.4)
Birth weight (g)	2489 ± 420
Birth‐weight percentile	10.0 ± 11.6
Birth weight < 10^th^ percentile	294 (62.8)
Birth weight < 3^rd^ percentile	136 (29.1)
Birth weight < 1^st^ percentile	34 (7.3)
Umbilical artery pH*	7.29 ± 0.08
Umbilical artery pH < 7.10	6/363 (1.7)
Apgar score at 5 min	9 (7–10)
Apgar score < 7 at 5 min	0 (0)
Stillbirth	1 (0.2)
NICU admission	108 (23.1)
Need for respiratory support at birth	32 (6.8)
Neonatal jaundice	81 (17.3)
Neonatal hypoglycemia	91 (19.4)
CAPO†	53 (11.3)
Length of neonatal hospitalization (days)	4 (1–42)

Data are given as mean ± SD, *n* (%), *n*/*N* (%) or median (range).

* Data were obtained from 363 cases.

† Composite adverse perinatal outcome (CAPO) was defined as stillbirth or a combination of at least two of the following: obstetric intervention due to intrapartum fetal distress (IFD), neonatal acidemia (umbilical artery pH < 7.10), birth weight < 3^rd^ percentile and transfer to neonatal intensive care unit (NICU).

BMI, body mass index; CS, Cesarean section; DM, diabetes mellitus; GA, gestational age; GDM, gestational diabetes mellitus; HDP, hypertensive disorder of pregnancy; ID, instrumental delivery; SVD, spontaneous vaginal delivery.

**Figure 2:**
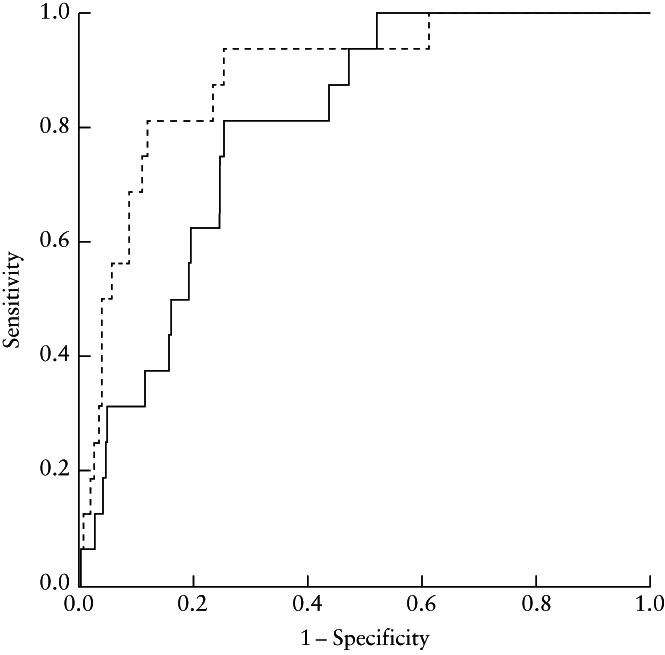
Receiver‐operating‐characteristics curves for prediction of composite adverse outcome (defined as stillbirth or at least two of the following: obstetric intervention due to intrapartum fetal distress, neonatal acidemia, birth weight < 3^rd^ percentile and transfer to the neonatal intensive care unit) by a model for estimating risk based on pregnancy characteristics, with (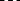) and without (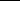) addition of estimated‐fetal‐weight percentile at diagnosis.

The results of logistic regression analysis evaluating the association between antenatal factors and perinatal outcome are shown in Table 2. EFW percentile at diagnosis and gestational age at delivery proved to be associated independently with the occurrence of CAPO (*P* = 0.01 and *P* < 0.01, respectively) and NICU admission (*P* < 0.01 for both), while mean UtA‐PI MoM > 95^th^ percentile at diagnosis was associated independently with obstetric intervention due to intrapartum fetal distress (*P* = 0.01). When assessing the baseline risk for CAPO using logistic regression analysis and a model including maternal age, booking body mass index, ethnicity, parity, smoking status, presence of comorbidities and gestational age at ultrasound examination (pretest probability), the addition only of EFW percentile at diagnosis resulted in a significant increase in the area under the ROC curve (0.889 (95% CI, 0.813–0.966) *vs* 0.803 (95% CI, 0.719–0.886); *P* = 0.02) (Figure 2).

**Table 2:** Multivariable logistic regression analysis of association of demographic, sonographic and antenatal parameters with occurrence of composite adverse perinatal outcome (CAPO), neonatal intensive care unit (NICU) admission and obstetric intervention due to intrapartum fetal distress (IFD), in 468 cases with late‐onset fetal growth restriction

	CAPO*		NICU admission		Obstetric intervention due to IFD	
Parameter	OR (95% CI)	*P*	OR (95% CI)	*P*	OR (95% CI)	*P*
EFW percentile at diagnosis	0.631 (0.440–0.904)	0.01	0.695 (0.542–0.890)	< 0.01	0.992 (0.879–1.121)	0.90
UA‐PI MoM > 95^th^ percentile at diagnosis	0.707 (0.076–6.563)	0.76	0.897 (0.144–5.598)	0.91	1.397 (0.282–6.922)	0.68
CPR MoM < 5^th^ percentile at diagnosis	1.191 (0.163–8.697)	0.86	0.738 (0.155–3.521)	0.70	0.598 (0.127–2.811)	0.52
Mean UtA‐PI MoM > 95^th^ percentile at diagnosis	1.445 (0.284–7.344)	0.66	1.612 (0.472–5.507)	0.45	4.373 (1.429–13.385)	0.01
Maternal age	1.063 (0.943–1.198)	0.32	0.989 (0.899–1.087)	0.82	0.981 (0.901–1.068)	0.67
Ethnicity	1.209 (0.278–5.252)	0.80	0.858 (0.161–4.563)	0.86	0.223 (0.026–1.946)	0.18
BMI at booking	0.901 (0.609–1.333)	0.60	1.055 (0.830–1.342)	0.66	0.908 (0.704–1.171)	0.46
BMI at term	1.153 (0.779–1.708)	0.48	0.949 (0.753–1.196)	0.66	1.174 (0.917–1.502)	0.20
Parity	1.215 (0.229–6.443)	0.82	0.528 (0.147–1.899)	0.33	0.859 (0.281–2.619)	0.79
Smoking	2.926 (0.444–19.272)	0.26	0.754 (0.148–3.833)	0.73	0.581 (0.107–3.147)	0.53
Comorbidity†	0.745 (0.136–4.082)	0.73	1.342 (0.377–4.776)	0.65	0.401 (0.088–1.820)	0.24
GA at delivery	0.477 (0.278–0.816)	< 0.01	0.327 (0.200–0.535)	< 0.01	0.767 (0.526–1.118)	0.17

* CAPO was defined as stillbirth or at least two of the following: obstetric intervention due to IFD, neonatal acidemia, birth weight < 3^rd^ percentile and transfer to the NICU.

† Presence of hypertensive disorder of pregnancy, diabetes mellitus/gestational diabetes or autoimmune disorder.

BMI, body mass index; CPR, cerebroplacental ratio; EFW, estimated fetal weight; GA, gestational age; MoM, multiples of the median; OR, odds ratio; UA‐PI, umbilical artery pulsatility index; UtA‐PI, uterine artery pulsatility index.

An EFW cut‐off corresponding to the 3.95^th^ percentile was found to discriminate between cases with and those without CAPO. EFW < 3.95^th^ percentile at diagnosis was associated with almost 3‐fold higher incidence of CAPO (19.7% *vs* 7.1%; *P* < 0.01), over 2‐fold higher rate of NICU admission (38.2% *vs* 15.4%; *P* < 0.01) and almost 2‐fold higher incidence of neonatal hypoglycemia (27.3% *vs* 15.4%; *P* < 0.01). Furthermore, EFW < 3.95^th^ percentile was associated with an increased rate of Cesarean section (48.4% *vs* 27.7%; *P* < 0.01) and obstetric intervention due to intrapartum fetal distress (12.7% *vs* 6.8%; *P* = 0.03), over 2‐fold higher frequency of delivery prior to 37 weeks' gestation (24.2% *vs* 10.3%; *P* < 0.01) and longer neonatal hospitalization (6 (2–42) *vs* 3 (1–37) days; *P* < 0.01) (Table 3). Overall, EFW < 3.95^th^ percentile at diagnosis proved to be a poor predictor of CAPO, yielding a sensitivity of 58.5% (95% CI, 44.1–71.9%), specificity of 69.6% (95% CI, 65.0–74.0%), positive predictive value of 19.8% (95% CI, 13.8–26.8%) and negative predictive value of 92.9% (95% CI, 89.5–95.5%).

**Table 3:** Maternal demographics, clinical characteristics and perinatal outcome of 468 cases with late‐onset fetal growth restriction, according to estimated‐fetal‐weight (EFW) percentile(p) at diagnosis

Parameter	EFW < 3.95^th^ p (*n* = 157)	EFW ≥ 3.95^th^ p (*n* = 311)	*P*
Maternal age (years)	32.9 ± 6.0	32.4 ± 5.6	0.46
BMI at booking (kg/m^2^)	22.0 ± 4.1	22.2 ± 4.1	0.74
BMI at delivery (kg/m^2^)	26.1 ± 4.8	26.4 ± 4.1	0.71
Ethnicity			0.43
Caucasian	139 (88.5)	263 (84.6)	
African	4 (2.5)	16 (5.1)	
Asian	11 (7.0)	21 (6.8)	
Other	3 (1.9)	11 (3.5)	
Nulliparous	113 (72.0)	215 (69.1)	0.53
Smoker	18 (11.5)	30 (9.6)	0.54
Comorbidity			0.84
HDP	9 (5.7)	19 (6.1)	
DM/GDM	11 (7.0)	19 (6.1)	
Autoimmune disorder	8 (5.1)	11 (3.5)	
GA at diagnosis (weeks)	34 + 1 ± 1 + 3	34 + 2 ± 1 + 4	0.30
UA‐PI MoM > 95^th^ p at diagnosis	21 (13.4)	15 (4.8)	< 0.01
CPR MoM < 5^th^ p at diagnosis	27 (17.2)	30 (9.6)	0.02
Mean UtA‐PI MoM > 95^th^ p at diagnosis	41 (26.1)	56 (18.0)	0.04
GA at last scan (weeks)	36 + 1 ± 1 + 4	37 + 0 ± 1 + 5	< 0.01
GA at delivery (weeks)	37 + 4 ± 1 + 3	38 + 5 ± 1 + 3	< 0.01
Delivery < 37 weeks	38 (24.2)	32 (10.3)	< 0.01
Delivery < 34 weeks	3 (1.9)	3 (1.0)	0.39
Mode of delivery			< 0.01
SVD	78 (49.7)	216 (69.5)	
ID	3 (1.9)	9 (2.9)	
CS	76 (48.4)	86 (27.7)	
Obstetric intervention due to IFD	20 (12.7)	21 (6.8)	0.03
Male neonate	83 (52.9)	134 (43.1)	0.05
Birth weight (g)	2212 ± 387	2629 ± 364	< 0.01
Birth‐weight p	8.8 ± 10.1	10.7 ± 12.3	0.09
Birth weight < 10^th^ p	103 (65.6)	191 (61.4)	0.38
Birth weight < 3^rd^ p	45 (28.7)	91(29.3)	0.89
Birth weight < 1^st^ p	12 (7.6)	22 (7.1)	0.82
UA pH*	7.29 ± 0.09	7.29 ± 0.08	0.79
UA pH < 7.10*	2/131 (1.5)	4/232 (1.7)	0.89
Apgar score at 5 min	9 (7–10)	9 (7–10)	< 0.01
Apgar score < 7 at 5 min	0 (0)	0 (0)	—
NICU admission	60 (38.2)	48 (15.4)	< 0.01
CAPO†	31 (19.7)	22 (7.1)	< 0.01
Need for respiratory support at birth	22 (14.0)	10 (3.2)	< 0.01
Intubation at birth	1 (0.6)	0 (0)	0.16
Neonatal jaundice	31 (19.7)	50 (16.1)	0.32
Neonatal hypoglycemia	43 (27.4)	48 (15.4)	< 0.01
Length of neonatal hospitalization (days)	6 (2–42)	3 (1–37)	< 0.01

Data are given as mean ± SD, *n* (%), *n*/*N* (%) or median (range).

* Data available in 363 cases.

† Composite adverse perinatal outcome (CAPO) was defined as stillbirth or combination of at least two of the following: obstetric intervention due to intrapartum fetal distress (IFD), neonatal acidemia, birth weight < 3^rd^ p and transfer to the neonatal intensive care unit (NICU).

BMI, body mass index; CPR, cerebroplacental ratio; CS, Cesarean section; DM, diabetes mellitus; GA, gestational age; GDM, gestational diabetes mellitus; HDP, hypertensive disorder of pregnancy; ID, instrumental delivery; MoM, multiples of the median; PI, pulsatility index; SVD, spontaneous vaginal delivery; UA, umbilical artery; UtA, uterine artery.

Approximately one‐third (162/468, 34.6%) of the included cases met the Delphi diagnostic criteria for late‐onset FGR. These cases showed an increased frequency of mean UtA‐PI MoM > 95^th^ percentile (29.6% *vs* 16.0%; *P* < 0.01), preterm delivery prior to 37 weeks (25.9% *vs* 9.2%; *P* < 0.01) and obstetric intervention (46.9% *vs* 32.1%; *P* < 0.01), earlier gestational age at delivery (37 + 4 ± 1 + 3 *vs* 38 + 5 ± 1 + 3 weeks; *P* < 0.01) and lower 5‐min Apgar score (*P* < 0.01), when compared with cases that did not fulfill the Delphi diagnostic criteria for late‐onset FGR. Furthermore, late‐onset FGR fetuses defined according to the Delphi criteria showed an increased incidence of perinatal adverse outcomes, including NICU admission (34.2% *vs* 17.3%; *P* < 0.01), need for respiratory support at birth (13.0% *vs* 3.6%; *P* < 0.01) and CAPO (17.9% *vs* 7.8%; *P* < 0.01) as well as longer neonatal hospitalization (5 (2–42) *vs* 3 (1–37) days; *P* < 0.01), when compared with cases that did not fulfill the Delphi diagnostic criteria for late‐onset FGR (Table S2).

## DISCUSSION

The findings of this cohort study demonstrate that non‐anomalous singleton pregnancies defined as late‐onset FGR, according to the study inclusion criteria, had a low frequency of maternal, perinatal and postnatal complications. Our data also show that EFW < 3.95^th^ percentile at the time of diagnosis of late‐onset FGR was the only ultrasound parameter associated independently with adverse perinatal outcomes, including prematurity and its potentially related complications, such as NICU admission, neonatal hypoglycemia and length of neonatal admission. Of note, the frequency of Cesarean section and obstetric intervention due to intrapartum fetal distress was shown to be almost 2‐fold higher among fetuses with EFW percentile < 3.95^th^ percentile. This finding is consistent with previous studies demonstrating a correlation between the risk of poor neonatal outcome, including stillbirth, and the actual EFW^21^, ^22^, ^23^. More specifically, the risk of adverse outcome has been shown to increase exponentially with decreasing EFW percentile^23^. Furthermore, our findings demonstrate an independent association between an abnormally raised mean UtA‐PI and obstetric intervention due to intrapartum fetal distress.

Unexpectedly, CPR MoM < 5^th^ percentile and mean UtA‐PI MoM > 95^th^ percentile at diagnosis did not prove to be associated independently with the occurrence of the primary outcome of this study. The finding of Doppler indicators of cerebral redistribution or UtA Doppler abnormalities has been suggested as a surrogate of clinical severity in the context of subclinical impairment of the placental function. On this basis, these parameters have been suggested to represent indication for elective delivery in the context of antenatally detected FGR^21^, ^22^. According to the recently published ISUOG guidelines, the use of CPR is endorsed in late‐onset FGR, while UtA Doppler assessment is not recommended for monitoring or determining the timing of delivery when a diagnosis of FGR is made^14^. Nonetheless, the findings of the present study and those reported by other research groups^20^, ^21^ support the notion that UtA Doppler abnormalities *per se* represent a risk factor for perinatal complications in the context of antenatally suspected FGR.

Several studies have demonstrated an association between reduced CPR and adverse outcome, including stillbirth, in fetuses with EFW < 10^th^ percentile^14^, ^21^, ^22^, ^37^, ^38^, ^39^, ^40^. The recently published findings of the TRUFFLE feasibility study demonstrated an association between composite adverse outcome and abnormal CPR close to delivery^16^. Consistent with those data, in this study we demonstrated an increase in the frequency of not only fetal but also maternal Doppler abnormalities with increasing severity of FGR. However, in our cohort, there was no association between CAPO and Doppler abnormalities at diagnosis. This unexpected finding could have resulted from the evaluation of Doppler parameters at diagnosis rather than close to delivery. While few or no data exist on longitudinal trends of maternal and fetal Doppler in late‐onset FGR^17^, ^18^, two recently published studies support the concept of progression of Doppler indicators of placental insufficiency across gestation in fetuses with EFW < 10^th^ percentile^16^, ^41^. A recent prospective study found a relationship between CPR and UtA Doppler abnormalities at diagnosis of late‐onset FGR and adverse outcome; however, it should be acknowledged that the criteria for the definition of FGR were not consistent with those adopted in the present study^42^. The concept that the diagnostic criteria impact on the occurrence of adverse outcome is not novel^43^ and is supported further by the subanalysis comparing the outcome of fetuses who met *vs* those who did not fulfill the Delphi diagnostic criteria for late‐onset FGR.

When looking at other perinatal outcomes, our data revealed an incidence of NICU admission of just above 1 in 4 cases, which is consistent with that reported in the studies of Figueras *et al*.^21^ and Madden *et al*.^44^. Prematurity, which has been recorded in approximately one‐fifth of cases in our cohort, is known to be related to an increased incidence of hypoglycemia^45^. However, more recent evidence suggests that FGR, associated with low body fat composition, represents the strongest risk factor for neonatal hypoglycemia^46^, ^47^. The low rate of need for respiratory support at birth is consistent with that recently reported by the TRUFFLE^16^ study and another research group^44^, while the 1‐in‐5 rate of hyperbilirubinemia confirms previous data reporting that FGR and prematurity represent independent risk factors^32^.

Albeit within the limitations of the adoption of different antenatal and postnatal growth charts, the unexpectedly high rate of neonates with birth weight above the 10^th^ percentile is likely to have resulted from the inclusion of fetuses considered at risk based on the longitudinal reduction of AC from mid‐trimester scan, which has been endorsed recently by the TRUFFLE group^16^. Of note, the 10^th^ percentile birth‐weight threshold was not associated with increased incidence of adverse perinatal outcome or with difference in the rate of Doppler abnormalities at diagnosis. The findings from a recent retrospective study of Meler *et al*.^41^ showed that EFW below the 10^th^ percentile in the absence of criteria of severity (i.e. fetal size below the 3^rd^ percentile or abnormalities of CPR or UtA Doppler) results in perinatal outcome comparable to that in normally grown neonates. In our cohort, the comparison between the fetuses identified as FGR and those with normal growth could not be undertaken, however, our results support the concept that the absence of criteria of severity is associated with improved perinatal outcome in the context of FGR.

To our knowledge, this is the first study providing information on perinatal outcome of late‐onset FGR based on sonographic findings at diagnosis. The main strength of this study is represented by the large number of included cases and its multicenter design involving three referral academic units. Of note, the participating centers had similar protocols for the perinatal management of late‐onset FGR.

On the other hand, the retrospective design is a major limitation of this study. Approximately one‐third of cases identified as late‐onset FGR were excluded due to incomplete/missing outcome or Doppler data. Another limitation is represented by the fact that there were no data concerning the indication for delivery other than for late‐onset FGR. More specifically, we reported a low incidence of HDP, diabetes mellitus/gestational diabetes and autoimmune disorders, potentially indicating elective delivery; however, we could not retrieve information on their co‐occurrence or on early delivery only for maternal indication. The antenatal care of FGR fetuses may be complicated by HDP^10^, ^11^, ^12^, ^13^, however, there is a paucity of data on the occurrence of HDP in pregnancies complicated by late‐onset FGR^16^, ^48^. Another limitation is that no data on longitudinal growth and Doppler up to delivery were considered in this study. Furthermore, different growth charts were adopted by the participating units. The choice of growth chart impacts the identification of late‐onset FGR, and we reported previously on the discrepancy of the growth charts adopted across different referral units in Italy^49^. Finally, cases were managed according to clinical and ultrasound parameters in the context of locally adopted protocols, which may account for a source of bias. Therefore, it should be acknowledged that no recommendations can be made based on the findings of the present study.

In conclusion, this study reports the maternal and perinatal outcomes of a selected cohort of late‐onset FGR fetuses identified on the basis of a biometric cut‐off or longitudinal growth criteria. While we report a low incidence of adverse perinatal events, we demonstrate that, when late‐onset FGR is diagnosed, EFW percentile at diagnosis represents the only sonographic parameter associated with composite adverse outcome and that abnormal UtA Doppler is associated with intrapartum fetal distress leading to obstetric intervention. These data can be used for antenatal counseling of prospective parents.

## Supporting information


**Table S1** Maternal demographics, clinical characteristics and perinatal outcome of cases with and those without birth weight < 10^th^ percentile

**Table S2** Maternal demographics, clinical characteristics and perinatal outcome of cases fulfilling *vs* those not fulfilling the Delphi diagnostic criteria for late‐onset fetal growth restriction (FGR) at diagnosis

## Data Availability

The data that support the findings of this study are available on request from the corresponding author. The data are not publicly available due to privacy or ethical restrictions.
