# Supplementary material for: Ultrasound prediction of adverse perinatal outcome at diagnosis of late‐onset fetal growth restriction
Source: Ultrasound Obstet Gynecol. 2022 Mar 3;59(3):342–9. doi: 10.1002/uog.23714 (PMC9313890; doi:10.1002/uog.23714)
Supplement: Supplementary file 1 — Table S1 Maternal demographics, clinical characteristics and perinatal outcome of cases with and those without birth weight < 10th percentile [file UOG-59-342-s001.docx]

|  | ***Birthweight <10 percentile***  ***N 294*** | ***Birthweight ≥10 percentile***  ***N 174*** | ***P*** |
| --- | --- | --- | --- |
| ***Maternal age, years***  ***Mean*** ± ***SD*** | 32.5 ± 5.9 | 32.7 ± 5.5 | 0.71 |
| ***Booking BMI, kg/m^2^***  ***Mean*** ± ***SD*** | 22.2 ± 4.3 | 22.0 ± 3.7 | 0.72 |
| ***BMI at delivery, kg/m^2^***  ***Mean*** ± ***SD*** | 26.5 ± 4.7 | 26.1 ± 3.6 | 0.51 |
| ***Ethnicity***  ***n (%)*** | Caucasian 253/294 (86.1%)  African 11/294 (3.7%)  Asian 19/294 (6.5%)  Other 11/294 (3.7%) | Caucasian 149/174 (85.6%)  African 9/174 (5.2%)  Asian 13/174 (7.5%)  Other 3/174 (1.7%) | 0.54 |
| ***Parity***  ***n (%)*** | Nulliparous 201/294 (68.4%) | Nulliparous 127/174 (73.0%) | 0.29 |
| ***Smoking***  ***n (%)*** | Smokers 27/294 (9.2%) | Smokers 21/174 (12.1%) | 0.32 |
| ***Comorbidity***  ***n (%)*** | HDP 15/294 (5.1%)  DM/GDM 19/294 (6.5%)  Autoimmune disorders 10/294 (3.4%) | HDP 13/174 (7.4%)  DM/GDM 11/174 (6.3%)  Autoimmune disorders 9/174 (5.2%) | 0.56 |
| ***Gestation at diagnosis, weeks^+days^***  ***Mean*** ± ***SD*** | 34^+2^ ± 1^+3^ | 34^+1^ ± 1^+4^ | 0.41 |
| ***Umbilical artery PI at diagnosis >95th percentile***  ***n (%)*** | 20/294 (6.8%) | 16/174 (9.2%) | 0.35 |
| ***Cerebroplacental ratio at diagnosis <5th percentile***  ***n (%)*** | 32/294 (10.9%) | 25/174 (14.4%) | 0.27 |
| ***Mean uterine artery PI at diagnosis >95th percentile***  ***n (%)*** | 60/294 (20.4%) | 37/174 (21.3%) | 0.83 |
| ***Gestation at last scan, weeks^+days^***  ***Mean*** ± ***SD*** | 36^+5^ ± 1^+4^ | 36^+6^ ± 1^+6^ | 0.74 |
| ***Gestation at delivery, weeks^+days^***  ***Mean*** ± ***SD*** | 38^+2^ ± 1^+3^ | 38^+3^ ± 1^+4^ | 0.40 |
| ***Delivery <37 weeks***  ***n (%)*** | 47/294 (16.0%) | 23/174 (13.2%) | 0.42 |
| ***Delivery <34 weeks***  ***n (%)*** | 4/294 (1.4%) | 2/174 (1.2%) | 0.84 |
| ***Mode of delivery***  ***n (%)*** | SVD 182/294 (61.9%)  ID 9/294 (3.1%)  CS 103/294 (35.0%) | SVD 112/174 (64.4%)  ID 3/174 (1.7%)  CS 59/174 (33.9%) | 0.64 |
| ***Obstetric intervention due to intrapartum fetal distress***  ***n (%)*** | 27/294 (9.2%) | 14/174 (8.0%) | 0.67 |
| ***Neonatal gender***  ***n (%)*** | Male 147/294 (50.0%) | Male 104/174 (59.8%) | 0.04 |
| ***Umbilical artery pH***  ***Mean*** ± ***SD***  ***n=363*** | 7.29 ± 0.09 | 7.29 ± 0.08 | 0.69 |
| ***Umbilical artery pH <7.10***  ***n=363*** | 4/230 (1.4%) | 2/133 (1.1%) | 0.87 |
| ***Apgar at 5 minutes***  ***Median (range)*** | 9 (7 – 10) | 9 (7 – 10) | 0.92 |
| ***Apgar <7 at 5 minutes***  ***n (%)*** | 0/294(0.0%) | 0/174 (0.0%) | - |
| ***NICU admission***  ***n (%)*** | 68/294 (23.2%) | 40/174 (23.0%) | 0.96 |
| ***Need for respiratory support at birth***  ***n (%)*** | 20/294 (6.8%) | 12/174 (6.9%) | 0.98 |
| ***Intubation at birth***  ***n (%)*** | 1/294 (0.3%) | 0/174 (0.0%) | 0.44 |
| ***Neonatal jaundice***  ***n (%)*** | 56/294 (19.1%) | 25/174 (14.4%) | 0.19 |
| ***Neonatal hypoglycemia***  ***n (%)*** | 58/294 (20.3%) | 33/174 (19.3%) | 0.80 |
| ***Length of neonatal hospitalization, days***  ***Median (range)*** | 4 (1 – 42) | 3 (1 – 37) | 0.15 |

SD: standard deviation

PI: pulsatility index

NICU: neonatal intensive care unit

BMI: body mass index

SVD: spontaneous vaginal delivery

ID: instrumental delivery

CS: cesarean section

HDP: hypertensive disorder of the pregnancy

DM: diabetes mellitus

GDM: gestational diabetes mellitus

*Defined by the combination of either stillbirth or at least two among obstetric intervention due to intrapartum fetal distress, neonatal acidemia (UA pH <7.10), birthweight <3rd centile and transfer to neonatal intensive care unit.
