# Supplementary material for: Ultrasound prediction of adverse perinatal outcome at diagnosis of late‐onset fetal growth restriction
Source: Ultrasound Obstet Gynecol. 2022 Mar 3;59(3):342–9. doi: 10.1002/uog.23714 (PMC9313890; doi:10.1002/uog.23714)
Supplement: Supplementary file 2 — Table S2 Maternal demographics, clinical characteristics and perinatal outcome of cases fulfilling vs those not fulfilling the Delphi diagnostic criteria for late‐onset fetal growth restriction (FGR) at diagnosis [file UOG-59-342-s002.docx]

|  | ***Fetuses fulfilling the Delphi diagnostic criteria for late-onset FGR***  ***N 162*** | ***Fetuses not fulfilling the Delphi diagnostic criteria for late-onset FGR***  ***N 306*** | ***P*** |
| --- | --- | --- | --- |
| ***Maternal age, years***  ***Mean*** ± ***SD*** | 33.1 ± 6.1 | 32.3 ± 5.6 | 0.18 |
| ***Booking BMI, kg/m^2^***  ***Mean*** ± ***SD*** | 22.2 ± 4.0 | 22.1 ± 4.1 | 0.70 |
| ***BMI at delivery, kg/m^2^***  ***Mean*** ± ***SD*** | 26.5 ± 4.4 | 26.2 ± 4.2 | 0.64 |
| ***Ethnicity***  ***n (%)*** | Caucasian 139/162 (85.8%)  African 7/162 (4.3%)  Asian 13/162 (8.0%)  Other 3/162 (1.9%) | Caucasian 263/306 (85.9%)  African 13/306 (4.2%)  Asian 19/306 (6.2%)  Other 11/306 (3.6%) | 0.66 |
| ***Parity***  ***n (%)*** | Nulliparous 118/162 (72.8%) | Nulliparous 210/306 (68.6%) | 0.34 |
| ***Smoking***  ***n (%)*** | Smokers 20/162 (12.3%) | Smokers 28/306 (9.2%) | 0.28 |
| ***Comorbidity***  ***n (%)*** | HDP 11/162 (6.8%)  DM/GDM 9/162 (5.6%)  Autoimmune disorders 6/162 (3.7%) | HDP 17/306 (5.6%)  DM/GDM 21/306 (6.9%)  Autoimmune disorders 13/306 (4.2%) | 0.89 |
| ***Gestation at diagnosis, weeks^+days^***  ***Mean*** ± ***SD*** | 34^+1^ ± 1^+3^ | 34^+3^ ± 1^+4^ | 0.17 |
| ***Mean uterine artery PI at diagnosis >95th percentile***  ***n (%)*** | 48/162 (29.6%) | 49/306 (16.0%) | <0.01 |
| ***Gestation at delivery, weeks^+days^***  ***Mean*** ± ***SD*** | 37^+4^ ± 1^+3^ | 38^+5^ ± 1^+3^ | <0.01 |
| ***Delivery <37 weeks***  ***n (%)*** | 42/162 (25.9%) | 28/306 (9.2%) | <0.01 |
| ***Delivery <34 weeks***  ***n (%)*** | 3/162 (1.9%) | 3/306 (1.0%) | 0.43 |
| ***Mode of delivery***  ***n (%)*** | SVD 86/162 (53.1%)  ID 6/162 (3.7%)  CS 70/162 (43.2%) | SVD 208/306 (68.0%)  ID 6/306 (2.0%)  CS 92/306 (30.1%) | <0.01 |
| ***Obstetric intervention due to intrapartum fetal distress***  ***n (%)*** | 19/162 (11.7%) | 22/306 (7.2%) | 0.10 |
| ***Neonatal gender***  ***n (%)*** | Male 85/162 (52.5%) | Male 132/306 (43.1%) | 0.05 |
| ***Umbilical artery pH***  ***Mean*** ± ***SD***  ***n=363*** | 7.29 ± 0.09 | 7.29 ± 0.08 | 0.99 |
| ***Umbilical artery pH <7.10***  ***n=363*** | 1/126 (0.8%) | 5/237 (2.1%) | 0.35 |
| ***Apgar at 5 minutes***  ***Median (range)*** | 9 (7 – 10) | 9 (7 – 10) | <0.01 |
| ***Apgar <7 at 5 minutes***  ***n (%)*** | 0/126 (0.0%) | 0/237 (0.0%) | - |
| ***NICU admission***  ***n (%)*** | 55/162 (34.2%) | 53/306 (17.3%) | <0.01 |
| ***Need for respiratory support at birth***  ***n (%)*** | 21/162 (13.0%) | 11/306 (3.6%) | <0.01 |
| ***Intubation at birth***  ***n (%)*** | 1/162 (0.6%) | 0/306 (0.0%) | 0.17 |
| ***Neonatal jaundice***  ***n (%)*** | 34/162 (21.1%) | 47/306 (15.4%) | 0.12 |
| ***Neonatal hypoglycemia***  ***n (%)*** | 37/162 (23.0%) | 54/306 (18.2%) | 0.23 |
| ***Composite adverse perinatal outcome****  ***n (%)*** | 29/162 (17.9%) | 24/306 (7.8%) | <0.01 |
| ***Length of neonatal hospitalization, days***  ***Median (range)*** | 5 (2 – 42) | 3 (1 – 37) | <0.01 |

SD: standard deviation

PI: pulsatility index

NICU: neonatal intensive care unit

BMI: body mass index

SVD: spontaneous vaginal delivery

ID: instrumental delivery

CS: cesarean section

HDP: hypertensive disorder of the pregnancy

DM: diabetes mellitus

GDM: gestational diabetes mellitus

*Defined by the combination of either stillbirth or at least two among obstetric intervention due to intrapartum fetal distress, neonatal acidemia (UA pH <7.10), birthweight <3rd centile and transfer to neonatal intensive care unit.
